# Origination and selection of ABCDE and *AGL6* subfamily MADS-box genes in gymnosperms and angiosperms

**DOI:** 10.1186/s40659-019-0233-8

**Published:** 2019-04-24

**Authors:** Gangxu Shen, Chih-Hui Yang, Chi-Yen Shen, Keng-Shiang Huang

**Affiliations:** 1 Department of Electrical Engineering, I-Shou University, Kaohsiung, Taiwan; 2 The School of Chinese Medicine for Post-Baccalaureate, I-Shou University, Kaohsiung, Taiwan; 3 College of Medicine, I-Shou University, Kaohsiung, Taiwan

**Keywords:** ABCDE gene, *AGL*6, MADS-box gene, Evolutionary events, Phylogenetic analysis

## Abstract

**Background:**

The morphological diversity of flower organs is closely related to functional divergence within the MADS-box gene family. Bryophytes and seedless vascular plants have MADS-box genes but do not have ABCDE or *AGAMOUS*-*LIKE6* (*AGL6*) genes. ABCDE and *AGL6* genes belong to the subgroup of MADS-box genes. Previous works suggest that the B gene was the first ABCDE and *AGL6* genes to emerge in plant but there are no mentions about the probable origin time of ACDE and *AGL6* genes. Here, we collected ABCDE and *AGL6* gene 381 protein sequences and 361 coding sequences from gymnosperms and angiosperms and reconstructed a complete Bayesian phylogeny of these genes. In this study, we want to clarify the probable origin time of ABCDE and *AGL6* genes is a great help for understanding the role of the formation of the flower, which can decipher the forming order of MADS-box genes in the future.

**Results:**

These genes appeared to have been under purifying selection and their evolutionary rates are not significantly different from each other. Using the Bayesian evolutionary analysis by sampling trees (BEAST) tool, we estimated that: the mutation rate of the ABCDE and *AGL*6 genes was 2.617 × 10^−3^ substitutions/site/million years, and that B genes originated 339 million years ago (MYA), CD genes originated 322 MYA, and A genes shared the most recent common ancestor with E*/AGL*6 296 MYA, respectively.

**Conclusions:**

The phylogeny of ABCDE and *AGL6* genes subfamilies differed. The *APETALA1* (*AP1* or A gene) subfamily clustered into one group. The *APETALA3/PISTILLATA* (*AP3/PI* or B genes) subfamily clustered into two groups: the *AP3* and *PI* clades. The *AGAMOUS/SHATTERPROOF/SEEDSTICK* (*AG/SHP/STK* or CD genes) subfamily clustered into a single group. The *SEPALLATA* (*SEP* or E gene) subfamily in angiosperms clustered into two groups: the *SEP1/2/4* and *SEP3* clades. The *AGL6* subfamily clustered into a single group. Moreover, ABCDE and *AGL6* genes appeared in the following order: *AP3/PI *→ *AG/SHP/STK *→ *AGL6*/*SEP*/*AP1.* In this study, we collected candidate sequences from gymnosperms and angiosperms. This study highlights important events in the evolutionary history of the ABCDE and *AGL6* gene families and clarifies their evolutionary path.

**Electronic supplementary material:**

The online version of this article (10.1186/s40659-019-0233-8) contains supplementary material, which is available to authorized users.

## Background

MADS-box genes played a crucial role in the emergence of flower structures during plant evolution [1, 2]. Moreover, the role of MADS-box genes in controlling flower morphogenesis makes them ideal genetic tools for studying the development of various flower structures [3]. The number of MADS-box genes in terrestrial plants is higher than in any other group of eukaryotes [4–7]. The term MADS-box gene is derived from four of the earliest recognized family members: *MINICHROMOSOME MAINTENANCE 1* (*MCM1*) from *Saccharomyces cerevisiae*, *AGAMOUS* (AG) from *Arabidopsis thaliana*, *DEFICIENS* (*DEF*) from *Antirrhinum majus*, and *SERUM RESPONSE FACTOR* (*SRF*) from *Homo sapiens* [4, 8]. An ancestral MADS-box gene was presumably duplicated before the most recent common ancestor (MRCA) of eukaryotes and evolved into two main clades, the *SRF*-like (type I) and *MEF2*-like (type II) MADS-box genes [9]. In Streptophyta (Charophyta algae and terrestrial plants), *MEF2*-like transcription factors (TFs) are often referred to as the MADS, intervening, keratin-like, and C-terminal type (MIKC-type) TFs, since their structures include a MADS (M)-domain that is followed by an intervening (I), a keratin-like (K), and a C-terminal (C) domains respectively [10, 11]. In terrestrial plants, the MIKC-type TFs form two main groups: the MIKC* and the MIKC^C^ type [12]. After these genes emerged, flowering plants diversified substantially during the Cretaceous period to become the largest plant group on earth [7]. Their remarkable evolutionary success was primarily due to the newly evolved reproductive structures and is similar to the success of gymnosperms which use seeds as a new propagation system [13]. The MIKC^C^ group can be further divided into 14 phylogenetic subfamilies [4, 6, 14], among which 10 are present in all angiosperms while 7 in all gymnosperms [6, 15]. Therefore, the appearance of MIKC^C^-type genes seems to be closely associated with the successful evolution of flowering plants.

Among MIKC^C^-type genes, the subgroups ABCDE and *AGAMOUS*-*LIKE 6* (*AGL6*) are key factors in flower development according to a proposed ABCDE model which suggests that combinations of various MADS-box genes determine the identity of flower organs [2, 8, 16]: A, B and C proteins function by interacting with E proteins which are necessary for all organ types [17]: A and E are present in sepals; A, B and E are present in petals; B, C and E are present in stamens; C and E are present in carpels [1, 3, 17–21]. However, some early studies in this field reported that the E gene is not expressed in sepals [22].

Related studies have reported MADS-box genes in gymnosperms [15, 23–27] and angiosperms [1, 3, 6, 18, 20, 28–30]. Selecting representative gymnosperm species from a range of families, including Gnetaceae (*G. gnemon*), Pinaceae (*P. abies*), Podocarpaceae (*P. macrophyllus*), Araucariaceae (*W. nobilis*), Sciadopityaceae (*S. verticillata*), Taxaceae (*T. baccata*), Cupressaceae (*C. japonica*) and Ginkgoaceae (*G. biloba*), allowed us to estimate a precise evolutionary timeline. In gymnosperms, some MADS-box genes are only expressed in reproductive organs, whereas most MADS-box genes, are expressed in both vegetative and reproductive organs [31]. This difference indicates that an increase in the number of MADS-box genes and the subsequent recruitment of some MADS-box genes as homeotic selector genes are important for the evolution of complex reproductive organs [32]. When selecting angiosperms, we included species from the three groups: (1) basal angiosperm (*A. trichopoda*) (2) monocots (*M. accuminata, O. sativa, Z. mays, and P. aphrodite*) (3) magnoliopsida and eudicots. Since magnoliopsida and eudicots is the largest group of angiosperm, we chose to include 14 typical species from the different families in this group, so that they would be useful for validating the evolutionary timeline. We considered choosing these seed plants (gymnosperms and angiosperms) for complete gene evolution of plants, which is of critical importance for the phylogenetic analysis. In related studies, bryophytes and seedless vascular plants do not have ABCDE or AGL6 genes but have MADS-box genes [33, 34].

Many studies have examined the origin of type II MADS-box genes accompanying the divergence of major plant lineages [35], some of which suggest that the type II MADS-box gene clades originated about 300 to 400 million years ago (MYA) [15, 35–38]. Molecular clock-based dating methods deduced that the B and C gene lineages originated 660 and 570 MYA respectively [39, 40], a period before the separation of the lineages that led to mosses, ferns, and seed plants. Alternatively, the type II MADS-box genes in the lineage that led to extant ferns may have evolved faster than those in the seed plant lineage, such that orthology between genes from ferns and seed plants can no longer be recognized [35]. Previous works suggest that the B gene was the first ABCDE and *AGL6* genes to emerge [15, 35–38] but there are no mentions about the probable origin time of ACDE and *AGL6* genes. Clarifying the probable origin time of ABCDE and *AGL6* genes is a great help for understanding the role of the formation of the flower, which can decipher the forming order of MADS-box genes in the future. In this study, we collected ABCDE and *AGL6* 381 protein sequences and 361 coding sequences from gymnosperms and angiosperms, in order to understand the evolutionary history of the ABCDE and *AGL6* genes.

## Results

### Identification of 381 ABCDE and *AGL6* genes

**Fig. 1:**
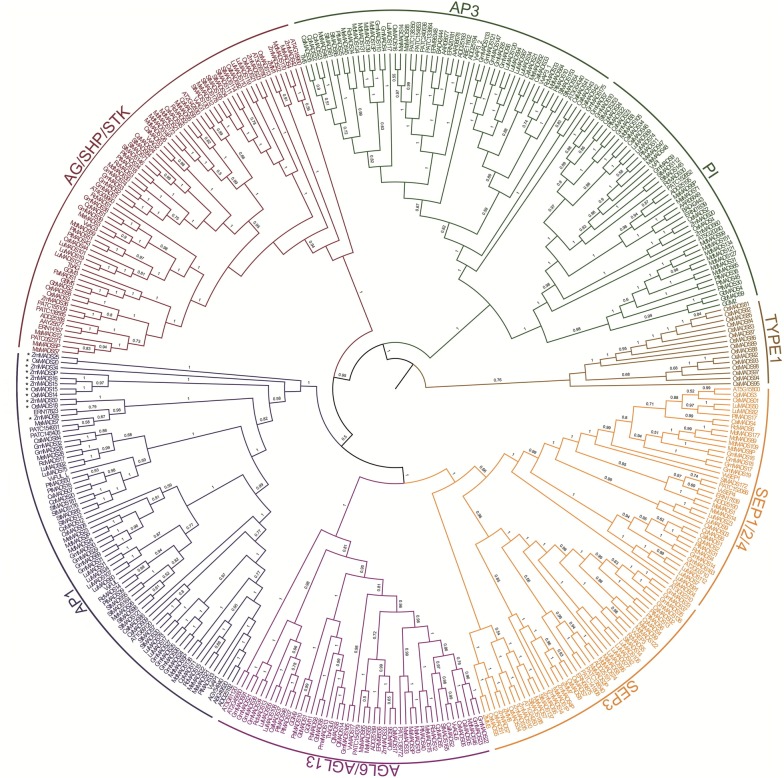
Phylogeny of the ABCDE and *AGL6* genes from 27 plant species and 381 classified protein sequences obtained using BEAST. The genes are indicated as follows: *AP1*, blue; *AP3/PI*, green; *AG/SHP/STK*, red; *SEP*, yellow; *AGL6/AGL13*, purple; and Type I MADS-box gene as an outgroup, brown. The Bayesian posterior probability values (numbers in black) are shown on the tree. Asterisks (*) represent Poaceae (*Oryza sativa: OsMADS14,15,18* and *20; Zea mays: ZmMADS3p, 8, 15, 16, 25, 34* and *50*)

To examine the evolutionary history of ABCDE and *AGL6* genes, we retrieved 381 sequences (Fig. 1, Table 1, Additional files 1, 2) from databases using known ABCDE and AGL6 protein sequences from *A. thaliana* and rice (*O. sativa*) as well as tomato MADS-box gene 6 (*TM6*) of *S. lycopersicum* as query sequences [2, 4, 6, 12, 29, 38, 41, 42] (Additional files 1, 2) in a BLAST search [43]. To verify the identities of the retrieved sequences before BLAST analyses, sequences were entered into the SMART to confirm the presence of basic MADS-box gene domains [44]. *AGL32* (B-sister genes) constitute a clade with a close relationship to class B genes [45]. Moreover, the B-sister and B genes arose 300–400 million years ago [45]. Therefore, we did not separate the B-sister and B genes in this study. The qualified sequences were aligned and included in the phylogenetic analyses. Sequences were arranged into subgroups according to the Bayesian phylogenetic tree in Fig. 1.

**Table 1:** Protein sequences of 27 plant species

No	Species	*AP1*	*AP3/PI*	*AG/SHP/STK*	*SEP*	*AGL6*
1	*Gnetum gnemon* (GGM)	−	+	+	−	+
2	*Picea abies* (Pa)	−	−	+	−	+
3	*Podocarpus macrophyllus* (Pma)	−	−	−	−	+
4	*Wollemia nobilis* (Wn)	−	−	−	−	−
5	*Sciadopitys verticillata* (Sv)	−	−	−	−	−
6	*Taxus baccata* (Tb)	−	−	+	−	+
7	*Cryptomeria japonica* (Cj)	−	−	−	−	+
8	*Ginkgo biloba* (Gb)	−	+	+	−	+
9	*Amborella trichopoda* (ERN, AAR, BAD, AAY, ERM)	+	+	+	+	+
10	*Musa accuminata* (Ma)	+	+	+	+	+
11	*Oryza sativa* (Os)	+	+	+	+	+
12	*Zea mays* (Zm)	+	+	+	+	+
13	*Phalaenopsis aphrodite* (PATC)	+	+	+	+	+
14	*Solanum lycopersicum* (TM)	N	+	N	N	N
15	*Solanum tuberosum* (St)	+	+	+	+	+
16	*Vitis vinifera* (Vv)	+	+	+	+	+
17	*Citrus sinensis* (Csi)	+	+	+	+	+
18	*Carica papaya* (Cp)	+	+	+	+	+
19	*Malus domestica* (Md)	+	+	+	+	+
20	*Arabidopsis thaliana* (AT)	+	+	+	+	+
21	*Cucumis sativus* (Cs)	+	+	+	+	+
22	*Glycine max* (Gm)	+	+	+	+	+
23	*Nelumbo nucifer* (ADD, AGY, ABG, ABE)	+	+	+	+	+
24	*Populus trichocarpa* (Pt)	+	+	+	+	+
25	*Linum usitatissimum* (Lu)	+	+	+	+	+
26	*Ricinus communis* (Rc)	+	+	+	+	+
27	*Manihot esculent* (Me)	+	+	+	+	+

The “N” means uncollected related sequence, (+) means have protein sequence and (−) means unrelated protein sequence in this study. Gymnosperms: number 1–8. Angiosperms: number 9–27

### Phylogenetic analysis of the ABCDE and *AGL6* genes

To depict the phylogenetic relationship among these 381 sequences, these genes were analyzed using Bayesian methods (Fig. 1). In previous studies, phylogenetic analysis of MADS-box genes in *Arabidopsis* and tomato was performed using the Bayesian methods for applied research [4, 46, 47]. In the present study, we used Bayesian method phylogenetic trees to sort individual sequences into subgroups (Fig. 1). The Bayesian method implemented in the Bayesian evolutionary analysis by sampling trees (BEAST) program was used to construct the phylogenetic tree (Fig. 1) representing the evolutionary relationship among all of the ABCDE and *AGL6* gene sequences, and to estimate the age of the ancestral node for each subgroup. Bayesian methods allow complex models of sequence evolution to be implemented [48]. According to Zhao et al. [49] the phylogenetic tree showing the relationships for the different functional gene clades of the MADS-box gene family ABCDE and *AGL6* genes is the major clades of MIKC^c^-type group. In this study, our first aim was to clarify the origin of ABCDE and *AGL6* genes.

### Variations in the number of ABCDE and *AGL6* genes in seed plants

The 381 ABCDE and *AGL6* sequences from 27 seed plants clustered into five subgroups: *APETALA1* (*AP1* or A gene, 74), *AP3/PISTILLATA* (*AP3/PI* or B genes, 101), *AG/SHATTERPROOF/SEEDSTICK* (*AG/SHP/STK* or CD genes, 75), *SEPALLATA* (*SEP* or E gene, 83), and *AGL6/AGL13* (*AGL 6* gene, 48) (Fig. 1, Additional files 1, 2). The highest number of ABCDE and *AGL6* genes in a flowering plant genome was observed in soybean (*Glycine max*) (45) and the highest number among the gymnosperms was observed in *G. biloba* (6). The flowering plant *N. nucifera* had the fewest ABCDE and *AGL6* sequences (11). The A/E/*AGL6* MADS-box genes formed a monophyletic clade (posterior probability [PP] = 0.5) that was larger (205) than the B (*AP3/PI*, 101) and CD (*AG/SHP/STK*, 75) clades (Fig. 1, Additional file 1).

### Evolutionary patterns of ABCDE and *AGL6* genes in plants

Previous works suggest that the B gene (*AP3/PI*) was the first ABCDE and *AGL6* genes to emerge [15, 35–38] (Fig. 1). Our results show that plants that arose since gymnosperms appeared approximately 305 MYA [50] have both B/CD and *AGL6* genes (Table 1). Moreover, the B-sister and B genes arose 300–400 million years ago [45]. Therefore, we propose that the reasonable time of the B gene (*AP3/PI*) originated about 300 to 400 MYA. Kishino et al. [51] have proposed Bayesian methods of estimating the dates associated with branch points in a phylogenetic tree. Using the BEAST program, we set the origin of the B gene (*AP3/PI*) to about 350 MYA, and used this as a calibration point to estimate the appearance times of the ACDE and *AGL6* genes. In this study, we use B gene as the arising standard, which is sound and is expected to yield accurate information, and use BEAST for estimating the possible arising time is feasible. We are hopeful that using the origin time of a specific gene will accurately predict the origin time of other genes. With the comprehensive analysis, it is critical importance of the time of evolution for ABCDE and *AGL6* genes.

#### *AP1* patterns

A-class genes are associated with sepal and petal development [17]. We found that only angiosperms possessed *AP1* genes (Table 1). According to our phylogenetic study (Fig. 1), the ancestral *AP1* diverged into one group. In monocots, the *AP1* genes seem to have undergone several duplication events. One duplication event appears to have occurred after the divergence of Poaceae (*O. sativa* and *Z. mays,* Fig. 1, Asterisks*) from the other monocots, resulting in the duplicates *OsMADS18/20* (Fig. 1) and *OsMADS14/15* (Fig. 1, Additional file 3). The highest number of *AP1* was observed in *S. tuberosum* and *G. max* (Additional file 1). These results suggest that *AP1* replicated frequently in higher angiosperms and the restriction of MADS-box gene expression to specific reproductive organs and the specialization of MADS-box genes as homeotic genes in angiosperms were crucial aspects of floral organ evolution. Consistent with previous reports [23, 27, 52], the *AP1* gene has not been observed in gymnosperms (Table 1). Since there is more completed genome data and in our research there are comprehensive sequence collections, we have newly discovered the sequences: ZmMADS16 and ZmMADS25 were in the *AP1* clade (Additional file 1, Asterisks*), which consistent with the findings of previous *AP1* genes studies [5, 18, 53–56].

**Fig. 2:**
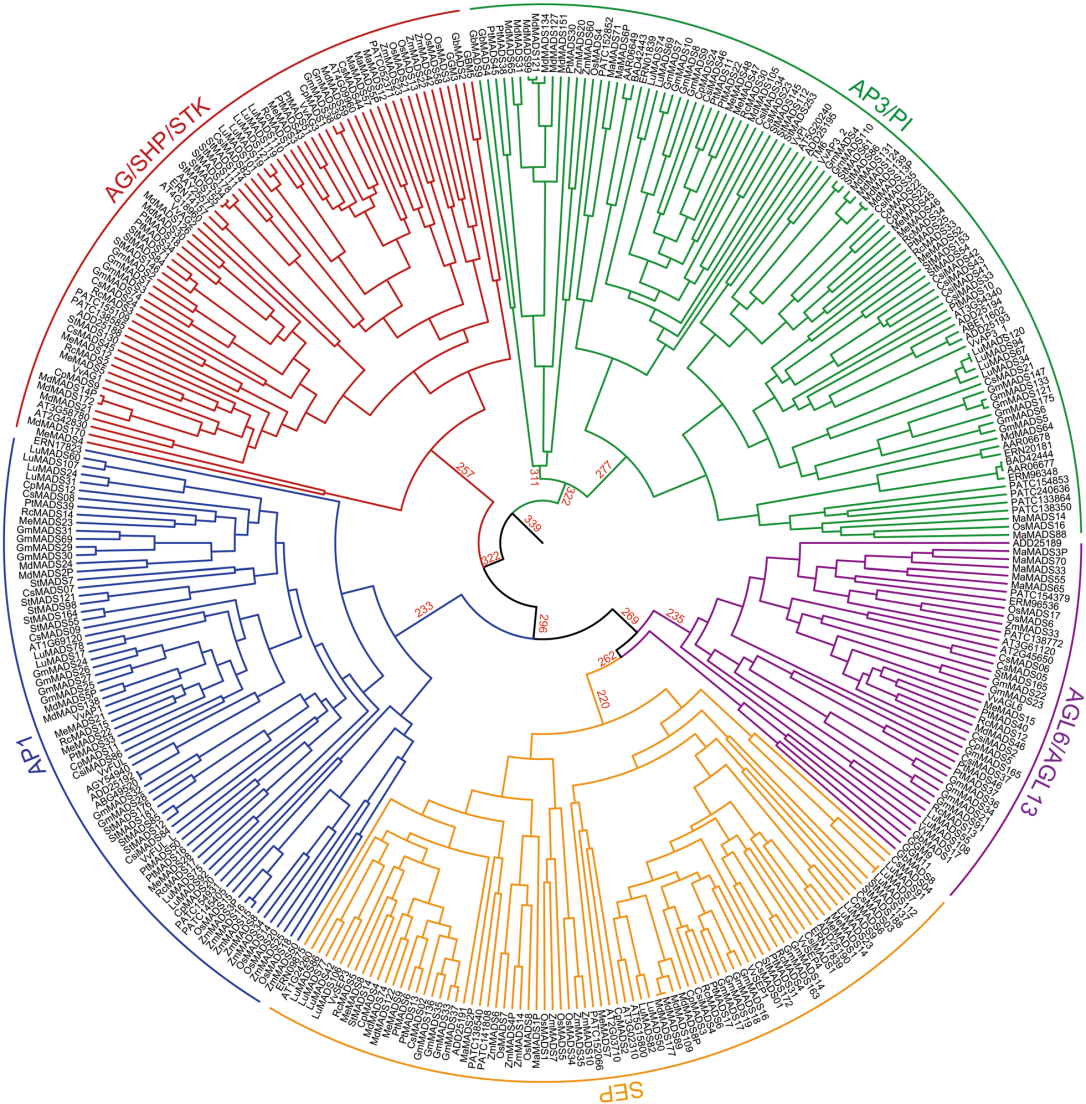
Phylogeny of the ABCDE and *AGL6* genes from 27 plant species and 361 classified coding sequences obtained using BEAST. The genes are colored as follows: *AP1*, blue; *AP3/PI*, green; *AG/SHP/STK*, red; *SEP*, yellow; and *AGL6/AGL13*, purple. The red values represent the probable origin and divergence time (million years ago, MYA) as calculated using BEAST of the *AP1*, *AP3/PI*, *AG/SHP/STK*, *SEP*, and *AGL6/AGL13* genes in the tree. *AP3/PI* originated 339 MYA; *AG/SHP/STK* originated 322 MYA; and *AP1*, *SEP*, and *AGL6/AGL13* originated 296 MYA. The divergence of *SEP* and *AGL6/13* occurred 269 MYA, and the divergence of *AP1* occurred 233 MYA

The clustering of *ERN17823* (Fig. 1) from the basal angiosperm *A. trichopoda* and *ZmMADS8* (Fig. 1) from *Z. mays* (PP = 0.79) indicates that *AP1* emerged before the divergence of *A. trichopoda* and then developed into the *AP1* of *Z. mays* with the fewest changes. It remains unknown why these genes have undergone large expansions in *S. tuberosum and G. max* (Additional file 1). After a gene duplication, selection pressure favors gene retention only if the loss of a gene reduces the fitness of the organism [38]. Regardless, many duplicated genes appear to be redundant, since their loss-of-function mutants do not result in any detectable deviations in phenotype; however, there are known cases in which purifying selection constrains the divergence between redundant genes [38]. To trace the possible time when the ABCDE and *AGL6* genes emerged, we used the BEAST tool and set the origin time of the B gene (*AP3/PI*) at 350 MYA to obtain a mutation rate estimate of 2.617 × 10^−3^ substitutions/site/million years for ABCDE and *AGL6* genes. We found that A gene (*AP1*) shared an MRCA with *SEP/AGL6/AGL13* 296 MYA and diverged into its own lineage 233 MYA (Fig. 2).

#### *AP3/PI* patterns

B-class genes play an important role in petal and stamen development [37]. Furthermore, the paleo*AP3*/*DEF* lineage produced two additional lineages in the eduicots known as eu*AP3* and *TM6* [22]. *Malus domestica* had the highest number of *AP3/PI* genes (13 sequences, Additional file 1). Angiosperms have more *AP3/PI* genes than gymnosperms, which may help form more complex reproductive organs. Unlike the variable number of *AP1* genes among dicots and monocots, there were no obvious difference in the number of *AP3/PI* genes between the monocots and dicots, although they have distinctly different second whorl structures (lodicule vs. petal) [15, 28]. In Fig. 1, we found that most *AP3* genes located in a single cluster comprising homologs of both eudicot and monocot and the *A. trichopoda* [49]. In the study, we have newly discovered the sequence: *PtMADS25* was in the *AP3/PI* clade (Additional file 1, Asterisks*), which consistent with the findings of previous *AP3/PI* genes studies [5, 18, 34, 52–57]. Among the 381 sequences from the 27 seed plants examined, the highest number of ABCDE and *AGL6* genes was observed in the *AP3/PI* with a total number of 101 (Additional file 1), suggesting that numerous *AP3/PI* gene expansions contribute to the evolution of reproductive organs. The MADS-box genes appear to have evolved mainly through gene duplication events, followed by neofunctionalization and subfunctionalization, or in certain cases, the pseudogenization of the duplicated gene [6].

Different to the distinct evolution pattern of *AP3* and *PI*, independent duplications of the B genes are being discovered in monocot and dicot species. These specific duplications are predicted to be associated with morphological innovation such as the highly derived petals of the Orchid family [58]. In dicots, some species have only a single *PI* gene, such as *C. papaya* (*CpMADS24*), *C. sativus* (*CsMADS23*), *R. communis* (*RcMADS30*) and *Vitis vinifera* (*VvPI*) (Fig. 1). Although the evolutionary patterns of the *PI* clade do not resemble the patterns of species expansion, *PI* genes from *G. gnemon, G. biloba, M. domestica,* and *P. trichocarpa* belong to one clade (PP = 0.98; Additional file 4). One possible explanation for this possibility is that the ancestors of gymnosperms, *G. gnemon* and *G. biloba* possessed the *PI* gene, whereas the other species lose this homolog during the evolutionary process. Another possibility is that horizontal gene transfer might have occurred, in which microorganisms or insects transferred the *PI* gene from *G. gnemon* or *G. biloba* into *M. domestica* and *P. trichocarpa*. This process can occur between closely related eukaryotic species [59], can mediate the massive transfer of chloroplast–nuclear genes [60], and the inter-species movement of chloroplasts under stress [61]. Evidence of this may found in the *PI* gene of *A. trichopoda* (*AAR06649, BAD42443* and *ERN01839*) (Fig. 1), a close relative of *M. accuminata, N. nucifera* and *P. aphrodite* (PP = 1; Additional file 4). Previously, the eu*AP3* lineage was a divergent paralogous group, only found in higher eudicots [62]. This study shows that *AP3* lineage formed a monophyletic clade in monocot (PP = 1; Fig. 1; *OsMADS16, MaMADS14, MaMADS88, PATC138350, PATC154853, PATC240636,* and *PATC133864*). Following examples set by other studies [15, 36–39], we used the BEAST tool to estimate when the B gene (*AP3/PI*) clade originated. We found that it arose 339 MYA and has a mutation rate of 2.617 × 10^−3^ substitutions/site/million years (Fig. 2), making it the first gene clade to have evolved that is involved in reproductive structure development (Fig. 2). Since gymnosperms appeared approximately 305 MYA [50], the *AP3/PI* genes might have appeared in the phase of evolution between seedless vascular plants and gymnosperms.

#### *AG/SHP/STK* patterns

CD-class genes are associated with stamen and carpel development [17]. As displayed in Fig. 1, the *AG/SHP/STK* genes formed a single clade (PP = 1). By contrast, the five gymnosperm genes *TbAG, GGM3, PaMADS1, GBM5*, and *GbMADS2* (Fig. 1) formed a well-supported clade (PP = 1; Additional file 5). Among the flowering plants, the highest number of *AG/SHP/STK* genes (9) was observed in *S. tuberosum*, whereas the lowest was observed in *N. nucifera* (1) (Additional file 1). Because of more completed genome data, we have newly discovered the sequences: *CsMADS24*, *CsMADS44*, *CsMADS45*, *GbMADS2* and *PtMADS34* were located in the *AG/SHP/STK* clade (Additional file 1, Asterisks*). Our research consistent with the findings of previous *AG/SHP/STK* genes studies [5, 18, 24, 26, 34, 52–56, 63–65].

BEAST analysis set the origin of the B genes (*AP3/PI*) to 350 MYA and yielded a mutation rate estimate of 2.617 × 10^−3^ substitutions/site/million years for the ABCDE and *AGL6* genes. Based on these data, we found that the CD (*AG/SHP/STK*) originated 322 MYA, shortly after the appearance of B gene (Fig. 2).

#### *SEP* patterns

E-class genes are associated with the formation of all floral organ types during reproductive development [17]. The *SEP* has been isolated from a few plants and its homologs in *Arabidopsis* include *SEP1*, *SEP2*, *SEP3*, and *SEP4*, [66]. Some analyses place *SEP1* and *SEP2* closer to *SEP4* than to *SEP3* [66] (Fig. 1), whereas other studies conclude that *SEP3* is the closest relative of *SEP1* and *SEP2* [15, 67, 68]. We found that only angiosperms possessed *SEP*, and that these genes clustered into two groups: *SEP1/2/4* and *SEP3* (Fig. 1). Our results also suggest *that SEP1* and *SEP2* are more closely related to *SEP4* than to *SEP3*. *SEP3* formed a monophyletic clade in monocot [69]. However, the two *SEP3* genes *PATC141808* and *PATC138540* of *P. aphrodite* unexpectedly fell outside of this clade (Fig. 1)*. SEP3* appears to have diverged more in the monocots than in the eudicots. In Fig. 1, most eudicot and monocot *SEP3* genes group as a distinct cluster [49]. In monocots, the *SEP3* lineage has undergone several duplication events (Fig. 1). One duplication event appears to have occurred after the divergence of Poaceae (*O. sativa* and *Z. mays*) from the remaining monocots, resulting in the duplicates *OsMADS7* and *OsMADS8* (Fig. 1). Our sampling was insufficient to determine whether this duplication is specific to the Poaceae or to all of the Poales [69]. This finding shows that the *SEP* genes of *S. tuberosum* and *M. esculenta* (*StMADS137, StMADS188* and *MeMADS7*) (Fig. 1) are closely related to the *SEP* genes in monocots. The *SEP1/2/4* of angiosperms clustered in a single clade (PP = 1; Additional file 6). In Fig. 1, some eudicot species (e.g. *G. max* and *M. domestica*) had several copies that formed species-specific clades that reside inside a well-supported *SEP1/2/4* clade (PP = 0.94; Additional file 6). The highest number of *SEPs* (11) was observed in *Linum usitatissimum* (Additional file 1). The flowering plants *A. trichopoda, M. accuminata*, and *N. nucifera* had the lowest number of *SEP* (Additional file 1); *SEP*s from these species underwent fewer duplications, implying that the E and ABCD genes are less involved in flower development in these species. The finding that basal angiosperms and monocots *M. accuminata* had less E gene expansion than did the other plants examined may indicate that the restriction of MADS-box gene expression to specific reproductive organs and the specialization of the MADS-box gene in the flowering plant lineage were crucial events in floral evolution. We have newly discovered the sequences: Z*mMADS6* and *ZmMADS7* were in the *SEP* clade (Additional file 1, Asterisks*), which consistent with the findings of previous *SEP* genes studies [5, 53, 54, 70].

The ABCDE and AGL6 genes had an estimated mutation rate of 2.617 × 10^−3^ substitutions/site/million years using BEAST analysis set the origin time of the B genes (AP3/PI) at 350 MYAWe found that *SEP* shared an MRCA with *AGL6/13* and *AP1* genes 296 MYA, and with *AGL6/13* 269 MYA (Fig. 2).

#### *AGL6/AGL13* patterns

The *AGL6*-like genes are associated with floral development in angiosperms [29] and with cone formation in gymnosperms [71]. The *AGL6*-like genes in monocots and eudicots play essential roles in floral development [41, 72]. The *Arabidopsis* genome contains two *AGL6* genes, namely *AGL6* and *AGL13* [66], suggesting a potential functional redundancy between these two genes. Schauer et al. [47] argued that *AGL6* and *AGL13* exhibit signs of subfunctionalization, with different expression patterns, regulatory sequences, and possible functions. *AGL6/13* and *SEP* genes have a high degree of sequence similarity and form sister clades in phylogenetic trees [30] (Fig. 1). As displayed in Fig. 1, the *AGL6/13* is categorized into one class (*AGL6/13*) in which genes of gymnosperms formed a well-supported clade (PP = 1; Additional file 7). Among the flowering plants, the highest number of *AGL6/13*s (7) was observed in *G. max*. Contrary to *SEP* genes which are only present in angiosperms, *AGL6/13* genes are ancient and widely distributed in gymnosperms and angiosperms (Additional file 1). In our research, there are comprehensive sequence collections, we have newly discovered the sequences: *CjMADS8*, *GmMADS91, GmMADS165, PaMADS10, PtMADS37, PtMADS 46* and *VvMADS17* were placed in the *AGL6/13* clade (Additional file 1, Asterisks*). Consistent with the findings of previous *AGL6/13 genes* studies [5, 18, 34, 52–55, 63–65, 70–73].

In this study, the *P. abies* gene *PaMADS8* was placed in the *AGL6*/*AGL13* subfamily (Fig. 1). *PaMADS8* (*DAL1*) was predicted to play a role in the transition from juvenile to adult plant (including the transition from reproductively incompetent to competent) [74]. This proposal was chiefly based on the observed expression pattern of *PaMADS8*; expression increased with the age of the tree and with the consecutive development of the vegetative structures within the tree. For instance, the relative expression of *PaMADS8* is highest in vegetative shoots in the apical part of the tree [74]. Both *PaMADS8* and other *AGL6* genes in gymnosperms are active in cones [52, 63]. These results revealed that *AGL6* gene redundancy and functional diversity also exist in gymnosperms. Using BEAST analysis and set the origin time of B genes (AP3/PI) at 350 MYA, and a mutation rate estimate of 2.617 × 10^−3^ substitutions/site/million years for ABCDE and *AGL6* genes. These results suggest that the *AGL6* family shared an MRCA with *SEP* and *AP1* genes 296 MYA (Fig. 2).

All calculations were implemented using codeml at PAML4.9. Different models were specified according to the software instruction. “np” refers to the number of parameters, “l = (ln L)” refers to the log value of the likelihood. The estimated parameters w refer to the dN/dS ratio. In the one-ratio model M0 and the Branch-specific two-ratio models, w (A), w (B), w (CD), w (CD), and w (AGL6) stand for the w ratios in the 27 plant species.

### Natural selection analysis

The assessment of synonymous (syn) and non-synonymous (non-syn) substitution ratios is important for understanding molecular evolution at the amino acid level [75]. To examine the intensity of natural selection acting on a specific clade, we examined the ratio (w) of non-syn substitutions to syn substitutions in our ABCDE and *AGL6* phylogeny. In this analysis, w < 1, w = 1, and w > 1 indicated purifying selection, neutral evolution, and positive selection, respectively. Based on our phylogeny, w assessments were conducted for five branches (w (A), w (B), w (CD), w (E), and w (*AGL6*) respectively). First, the branch-specific likelihood model [76] was applied to the ABCDE and *AGL6* data. As shown in Table 2, the one-ratio model revealed a w value of 0.29953, which is well below 1. This indicates a strong purifying selection pressure on the entire MADS-box gene family [77]. MADS-box proteins may be responsible for simultaneous increases in the ratio of nonsynonymous to synonymous substitutions early in angiosperm history and following concerted duplication events [77]. In contrast to the patterns of positive selection of *AP3/PI* reported by Hernandez–Hernandez et al. [37], however, we did not detect positive selection on ABCDE and *AGL6* genes within MADS box subfamilies. We used 361 classified coding sequences for our analysis of natural selection, and this would not affect the real relationships among these subfamilies as determined using ABCDE and *AGL6* genes to establish the phylogenetic tree. In the study, our results indicate that purifying selection has played an important role in the evolution of these MADS box gene subfamilies throughout seed plant history [77]. For the two-ratio model, when A, B, CD, E and *AGL6* genes were set as the out-branch, no significant differences were detected for any of the target genes (2∆*l*  = 0 or 2∆*l *= *1.6, p *=* 0.2059*, *df *=* 1*), suggesting that the evolutionary rates of A, B, CD, E, and *AGL6* genes are not significantly different from each other. We have also tested the multiple ratio models including the five-ratio model w (A) ≠ w (B) ≠ w (CD) ≠ w (E) ≠ w (AGL6). The results showed that this model is not better than the two-ratio model w (B) = w (CD) ≠ w (AGL6) = w (A) = w (E). Our analyses support B and CD have underwent significantly different selection pressure from A, E and *AGL6.* Regarding the biased sampling of the MIKC^c^-type genes, since the dN/dS ratio is a pair-wise characteristic, the w values we calculated for each branch represent the average dN/dS value for each specified branch. In our analyses, we specified CD genes altogether as a single branch and calculated their average dN/dS value. The current sampling of the MIKC^c^-type genes is sufficient and representative for the assessment of the selection pressure in this branch.

**Table 2:** Natural selection test of plant MADS-box genes

Model	Np	l = lnL	Estimates of parameters
M0: one-ratio			
w (A) = w (B) = w (CD) = w (E) = w (AGL6)	1	− 57551.8	w (A) = w (B) = w (CD) = w (E) = w (AGL6) = 0.29953
Branch-specific two-ratio models			
w (B) = w (CD) = w (E) = w (AGL6) ≠ w (A)	2	− 57551.8	w (B) = w (CD) = w (E) = w (AGL6) = 0.29953 w (A) = 2.13680
w (A) = w (CD) = w (E) = w (AGL6) ≠ w (B)	2	− 57551.0	w (A) = w (CD) = w (E) = w (AGL6) = 0.29920 w (B) = 301.74451
w (A) = w (B) = w (E) = w (AGL6) ≠ w (CD)	2	− 57551.8	w (A) = w (B) = w (E) = w (AGL6) = 0.29953 w (CD) = 2.12982
w (A) = w (B) = w (CD) = w (AGL6) ≠ w (E)	2	− 57551.8	w (A) = w (B) = w (CD) = w (AGL6) = 0.29954 w (E) = 2.12305
w (A) = w (B) = w (CD) = w (E) ≠ w (AGL6)	2	− 57551.8	w (A) = w (B) = w (CD) = w (E) = 0.29953 w (AGL6) = 2.11723
w (B) = w (CD) ≠ w (AGL6) = w (A) = w (E)	2	− 55162.3	w (AGL6) = w (A) = w (E) = 0.30546 w (B) = w (CD) = 0.00010
Five-ratio models			
w (A) ≠ w (B) ≠ w (CD) ≠ w (E) ≠ w (AGL6)	5	− 55190.1	w (A) = 0.30530, w (B) = 1.74205, w (CD) = 0.78636, w (E) = 0.64375, w (AGL6) = 1.69816

## Discussion

### B-class genes (*AP3/PI*) two possible evolutionary pathways

**Fig. 3:**
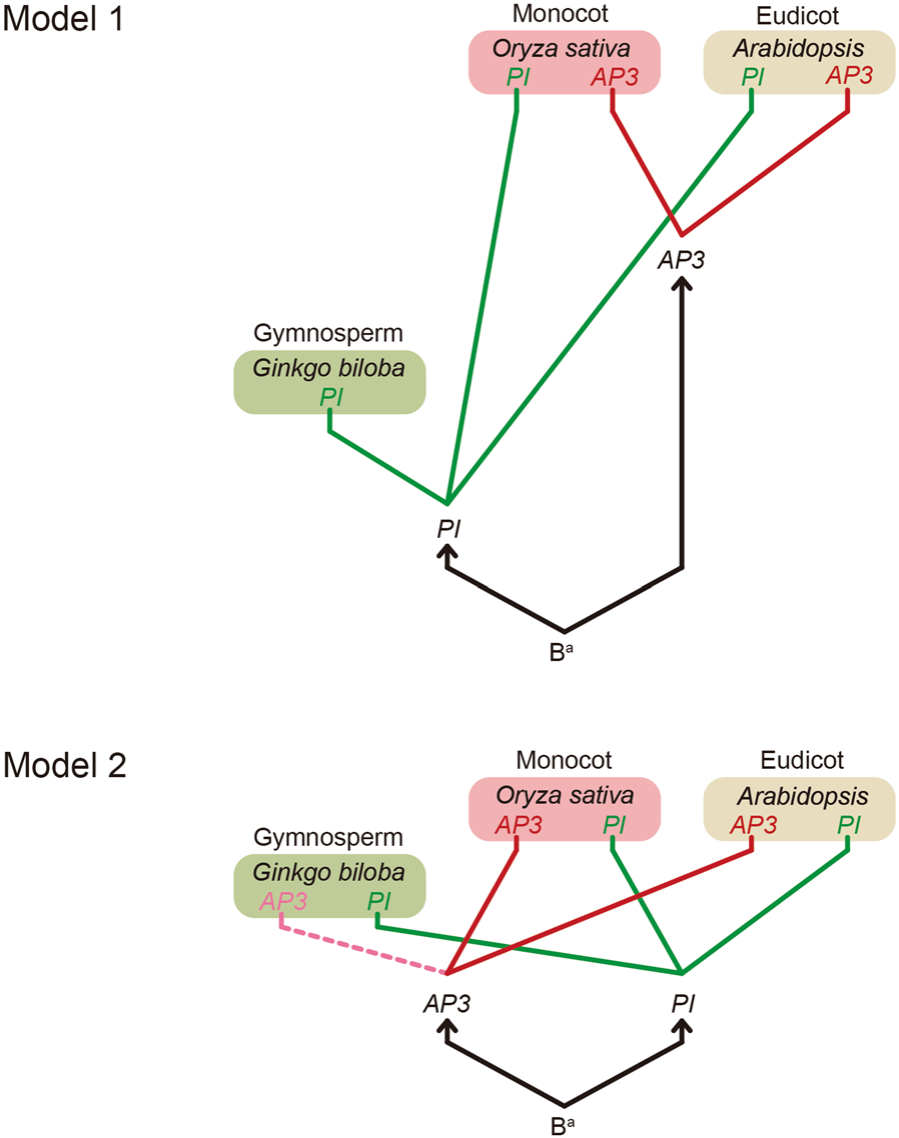
Evolution of B-class genes (*AP3/PI*) and their roles in *Arabidopsis thaliana*, *Oryza sativa,* and *Ginkgo biloba.* Model 1: The progenitor of the B-class gene (B^a^) primarily evolved from a *PI* lineage and generated the *AP3* and *PI* lineages through duplication. However, it evolved the *PI* in gymnosperm before duplication of the B-class gene generated the AP3 lineage in the angiosperm. *Arabidopsis* represents *Arabidopsis thaliana*. *Arabidopsis thaliana* and *Oryza sativa* have *AP3* and *PI*. Other angiosperms with the B-class gene also possess *AP3* and *PI*. Model 2: A duplication generated the ancestral (B^a^) *AP3* and *PI* lineages and a subsequent duplication; however, the *AP3* lineage was subsequently lost in gymnosperms. *Arabidopsis* represents *Arabidopsis thaliana*. *Arabidopsis thaliana, Oryza sativa* and other angiosperms with B-class genes have *AP3* and *PI*

Bryophytes and seedless vascular plants do not have ABCDE or *AGL6* genes [33, 34]. The gymnosperms have *PI* genes (*GGM2, GbMADS4* and *GbMADS9;* Fig. 1) but no *AP3* gene. Our study suggests that the *PI* clade probably evolved earlier than the *AP3* clade, and that the formation of gymnosperm cones depended on the presence of a *PI* ancestor. Therefore, the phylogeny of B-class genes lead us to infer two possible evolutionary pathways (Fig. 3). (1) The progenitor of the B gene (B^a^) first evolved through the *PI* lineage and then generated the *AP3* and *PI* lineages, since only the *PI* lineage was maintained in gymnosperms before the duplication of the B gene generated the *AP3* lineage in angiosperm. (2) An ancient duplication may have generated the ancestral (B^a^) *AP3* and *PI* lineages, and the *AP3* lineage was lost in gymnosperms after a subsequent duplication. Since the initial duplication that generated the paralogous *PI* and *AP3* lineages predates the monocot and dicot division, monocots have both clades [58]. Therefore, a more complete collection of sequences in diverse species would provide a clearer understanding of B gene (*AP3/PI*) duplication.

### *AP3/PI* and *AG/SHP/STK* evolved earlier

Previously, Kim et al. [78] assumed that the B gene evolved relatively earlier than other flower identity genes. *AG*, *AGL6* and *DEF *+ *GLO* (B genes) were present in the MRCA of angiosperms and gymnosperms approximately in 300 MYA [15]. Although some ancestral genes might have specialized 300 MYA in the development of male reproductive organs (*DEF/GLO*-like genes), female reproductive organs (*GGM13*-like genes) [79] or both (*AG* gene, *AGL2*, and *AGL6*-like genes), all of the MADS-box gene types were highly diversified before the establishment of the ovule approximately 300–400 MYA [36]. In this study, gymnosperms possessed *AP3/PI*. We estimated that *AP3/PI* originated 339 MYA (Fig. 2). Hence, we suggest that *AP3/PI* evolved before the appearance of gymnosperms, which in turn appeared approximately 305 MYA [50]. *AP3/PI* may have an ancestral function that is realized in extant gymnosperms in distinguishing male cones (form when the B gene is expressed) from female cones (form when the B gene is not expressed) [63]. The B gene is involved in the development of petals and stamens in angiosperms and male cones in gymnosperms [63, 80]. Kim et al. [78] also show that *AP3/PI* duplication occurred shortly after the divergence of extant gymnosperms and angiosperms, which is accordingly before the age of the oldest flowering plant fossils. This implies that the joint expression of *AP3* and *PI* may not have resulted in the immediate formation of petals, which they presently control in the development of extant angiosperms. Therefore, the earliest angiosperms may have been biochemically flexible in their B gene function [78].

We estimate that *AG/SHP/STK* (CD genes) evolved 322 MYA, shortly after the appearance of *AP3/PI* (B genes) (Fig. 2). The C gene has a single function in reproductive organ development and the mechanisms controlling its expression domain and evolution were key factors in the emergence of flowering plants [81]. Jager et al. [64] show that *GBM5* (CD gene; Additional file 1) is expressed in the early stages of developing male and female organs, and persists in the female gametophyte. Parallel expression patterns have been detected for the orthologues of *GBM5* in coniferophytes: *DAL2* (*PaMADS1*, CD gene; Additional file 1) in *P. abies* and *SAG1* in *Picea mariana*. *DAL2* and *SAG1* are expressed in male and female cones, but a gradual diminution was observed during the maturation of male cone, whereas female cones experience the development which maintained a great level of expression in respect of ovule maturation [82]. In contrast to this, the expression of *GGM3* (CD gene; Additional file 1) from *G. gnemon* persists in both male and female reproductive units in the late developmental stages [63]. In gymnosperms, some MADS-box genes are only expressed in reproductive organs, whereas most MADS-box genes, are expressed in both vegetative and reproductive organs [31]. This difference indicates that an increase in the number of MADS-box genes and the subsequent recruitment of some MADS-box genes as homeotic selector genes are important for the evolution of complex reproductive organs [32]. The expansion of the MIKC gene family in seed plants and increased plant complexity seem to be correlated [83]. Hence, CD genes (*AG/SHP/STK*) appeared to have evolved soon after the B genes (*AP3/PI*), and their emergence promoted reproduction in plants.

In different gymnosperms, *AG*-like genes are expressed in male and female reproductive organs, which may represent the ancestral state of gene expression [63]. These genes were suggested to function ancestrally in male and female cone formation, and in distinguishing them from the nonreproductive organs [63]. Several angiosperm *AG*-like genes probably have an ancestral function in specifying both male and female reproductive organs and have derived functions that are restricted to the stamen or pistil [84]. In male cones, microsporangia, which contain the pollen, develop at the base of the microsporophylls. By contrast, in female cones, uncovered ovules develop on the surface of megasporophylls, instead of being enclosed in a gynoecium [20]. Sporophylls are modified leaf-like organs that are the gymnosperm structures most closely related to carpels [20]. Therefore, angiosperm flowers and gymnosperm cones are homologous. Gymnosperms express B and CD genes, and the wide distribution of these genes throughout the gymnosperms shows that these genes were present when the gymnosperms first appeared. Since the B and CD genes are responsible for the formation of reproductive organs, the B and CD genes may have evolved before the A/E/*AGL6* superclade (Fig. 2).

### The A/E/*AGL6* superclade evolved soon after *AG/SHP/STK*

In our study, two *G. gnemon* genes (*GGM9* and *GGM11*) were placed in the *AGL6*/*AGL13* subfamily (Additional file 1). Both of these *G. gnemon* genes are expressed in both male and female reproductive cones, but not in vegetative leaves [63]. Katahata et al. [71] showed that *CjMADS14* (*AGL6*; Additional file 1) of *C. japonica* was expressed chiefly in male and female strobili. Together, these and other results suggest that *AGL6* is associated with reproduction [85, 86] and cone formation [71]. Our phylogenetic analysis revealed that *AGL6* family members are closely related to *SEP* with the MRCA occurring 296 MYA (Fig. 2). *Arabidopsis SEP* and *AGL6* genes were found to activate the expression of B and C genes [85]. Moreover, no *SEP* was found in gymnosperms (Table 1) [25]. *AGL6* does not directly influence floral structures; however, it is critical for the reproductive abilities of both gymnosperms and angiosperms. Thus, we propose that *AGL6* may have evolved after the formation of certain essential reproductive organs (e.g. flowers and cones) to aid in the formation of more complete reproductive structures in plants. *AGL6* genes may have played an important role in the evolution of unique flower features [86]. Li et al. [72] proposed that both *SEP* and *AP1* in angiosperms were derived from the common antecedent of *AGL6* within two duplication events, and another duplication event of *AGL6* genes likely arose before the derivation of grass. These findings suggest that *AGL6* genes may act in an ancient and conserved floral development pathway. Comparative analyses of spatiotemporal expression patterns of *AGL6* or genetic analyses on mutants are warranted to elucidate the functional redundancy of *AGL6* in lateral organ development and flowering [85]. Yoo et al. [87] showed that AGL6 regulates the transcription of two critical flowering-time regulators: *FLC* and *FT*. Moreover, AGL6 further enhanced *FT* expression in the absence of the *FLC* function, suggesting that AGL6 regulates *FT* independently of *FLC* [87]. Thus, based on the concept of evolution, the plant must flower at an appropriate time, which implies that *AGL6* have emerged before *AP1*.

Wang and Melzer [88] suggested that the AG-like protein GGM3 (CD protein; Additional file 1) can form homotetramers and even more stable heterotetramers with the DEF/GLO-like protein GGM2 (B protein; Additional file 1). Therefore, the capacity of gymnosperm MADS-domain proteins to produce multimeric complexes is similar to their angiosperm counterparts. However, in contrast to angiosperms, multimeric complex formation does not depend on the E proteins orthologues, and *SEP* genes have not yet been identified in any gymnosperm [88]. Furthermore, the genomes of most gymnosperms examined in our study possess *AGL*6, but not *SEP* (Table 1). Consequently, *AGL*6 and *SEP* genes estimated to have originated at a similar point in time (Fig. 2) but we hypothesize that *AGL*6 evolved before the *SEP* genes. According to our phylogenetic analysis and hypothesis, the ABCDE and *AGL6* genes may appear in the following order: *AP3/PI *→ *AG/SHP/STK *→ *AGL6 *→ *SEP *→ *AP1*.

## Conclusions

We assembled a comprehensive dataset of ABCDE and *AGL6* genes of representative species from gymnosperms and angiosperms as well as used it to construct the phylogeny of plant ABCDE and *AGL6* genes. We have newly discovered the sequences: *AP1* (*ZmMADS16* and *ZmMADS25*); *AP3/PI* (*PtMADS25*)*; AG/SHP/STK* (*CsMADS24, CsMADS44, CsMADS45, GbMADS2,* and *PtMADS34*)*; SEP* (*ZmMADS6* and *ZmMADS7*)*; AGL6/13* (*CjMADS8, GmMADS91, GmMADS165, PaMADS10, PtMADS37, PtMADS46* and *VvMADS17*) and these newly discovered the sequences are important for estimating the time of origin for ABCDE and *AGL6* genes, which can help to compensate the insufficient source of former researches. The phylogeny of ABCDE and *AGL6* genes subfamilies differed. The *AP1* subfamily clustered into one group. The *AP3/PI* subfamily clustered into two groups: the *AP3* and *PI* clades. The *AG/SHP/STK* subfamily clustered into a single group. The *SEP* subfamily in angiosperms clustered into two groups: the *SEP1/2/4* and *SEP3* clades. Finally, the *AGL6/13* subfamily clustered into a single group. The ABCDE and *AGL6* genes appeared in the following order: *AP3/PI* genes originated 339 MYA, *AG/SHP/STK* genes originated 322 MYA, *AP1* genes shared the MRCA with *AGL6*/*SEP* 296 MYA, and *AGL6*/*SEP* diverged in 269 MYA. Moreover, the phylogeny of B-class genes lead us to infer two possible evolutionary pathways. (1) The progenitor of the B gene (B^a^) first evolved through the *PI* lineage and then generated the *AP3* and *PI* lineages. (2) An ancient duplication may have generated the ancestral (B^a^) *AP3* and *PI* lineages, and the *AP3* lineage was lost in gymnosperms after a subsequent duplication. This study highlights important events in the evolutionary history of the ABCDE and *AGL6* gene families and clarifies their evolutionary path.

## Methods

### Identifying MADS-box sequences

To obtain sequences of model organisms, species-specific databases, including the *O. sativa* database (http://rice.plantbiology.msu.edu/), the *A. thaliana* database (http://www.arabidopsis.org/), and the *P. aphrodite* database (http://orchidstra.abrc.sinica.edu.tw). The sequences for *A. trichopoda* were obtained from the NCBI (http://www.ncbi.nlm.nih.gov/) and UNIPROT (http://www.uniprot.org/uniprot/). The sequences for angiosperms were obtained from the Gramene (http://www.gramene.org/) and Phytozome (http://www.phytozome.net/) databases. The sequences for gymnosperms were obtained from NCBI, Phytozome, and UNIPROT. Some databases such as PANTHER (http://www.pantherdb.org/), PGDD (http://chibba.agtec.uga.edu/duplication/), and Ensembl Plants database (https://plants.ensembl.org/index.html) are references of this research. Known ABCDE and *AGL6* protein sequences from *A. thaliana* and *O. sativa* as well as the *TM6* sequence of tomato (*S. lycopersicum*) were used as the query sequences (Additional files 1, 2) [2, 4, 6, 12, 29, 38, 41, 42] for BLASTP [43]. We applied an E-value cutoff of less than 10^−10^ for protein similarity.

### Confirming MADS-box sequences

First, all of the sequences obtained through BLAST were entered into SMART (http://smart.embl-heidelberg.de/) to confirm the presence of the basic domains from the MADS-box gene [44]. MIKC-type structures include M-domain that is followed by I, K, and C domains respectively [10, 11]. Subsequently, qualified sequences were aligned and subjected to phylogenetic tree analysis to determine their subfamily affiliations.

### Building the alignment and phylogenetic trees

The amino acid sequences were aligned using the program MUltiple Sequence Comparison by Log-Expectation (MUSCLE) in MEGA6. In addition, BEAST 2.2.1 was used to construct Bayesian phylogenies [4, 89]. The BEAST analysis was performed using a JTT substitution model and a Yule priors model. The stationary distribution of the MCMC chains and the convergence of runs were monitored using Tracer (v.1.6) to determine the appropriate MCMC chain length such that the effective sample size of every parameter was larger than 200 as recommended. Tree pictures were generated using TreeAnnotator (v. 1. 8. 2), with first 20000 trees discarded as burn-in. Trees were visualized using Figtree (v. 1. 4. 2).

### Natural selection analysis

Natural selective pressure on plant ABCDE and *AGL6* genes were examined by measuring the ratio of non-synonymous to synonymous substitutions (dN/dS = w). Codon-based maximum likelihood estimates of w was performed using codeml in PAML4.9 [90]. Multiple-alignment of conserved domain sequences for those identified plant ABCDE and *AGL6* genes were carried out using ClustalW2 [91]. Significant insertions and gaps were removed manually. To facilitate the input data requirements of codeml, an additional Maximum Likelihood tree was constructed using a smaller data set where the ABCDE and *AGL6* genes with no identifiable conserved domain sequences were removed. The subtree including plant ABCDE and *AGL6* genes was used in codeml. Branch pattern specification was implemented using Treeview1.6.6 (http://taxonomy.zoology.gla.ac.uk/rod/treeview.html). Five target clades were specified based on the present phylogenetic analysis: A, B, CD, E and *AGL6* genes. The w values for these clades were represented as w (A), w (B), w (CD), w (E), and w (AGL6) respectively. Nested likelihood ratio tests were performed to assess the significance of the model under different hypotheses: (w (B) = w (CD) = w (E) = w (AGL6) ≠ w (A), w (A) = w (CD) = w (E) = w (AGL6) ≠ w (B), w (A) = w (B) = w (E) = w (AGL6) ≠ w (CD), w (A) = w (B) = w (CD) = w (AGL6) ≠ w (E), w (A) = w (B) = w (CD) = w (E) ≠ w (AGL6), and w (B) = w (CD) ≠ w (AGL6) = w (A) = w (E)). The corresponding p values were calculated using the online tool at http://graphpad.com/quickcalcs/PValue1.cfm.

## Additional files

**Additional file 1.** Sequence numbers of 27 plant species.

**Additional file 2.** Txt files of 27 species and 381 sequences.

**Additional file 3.** The *AP1* genes in Bayesian tree and the Bayesian posterior probability values in tree.

**Additional file 4.** The *AP3/PI* genes in Bayesian tree and the Bayesian posterior probability values in tree.

**Additional file 5.** The *AG/SHP/STK* genes in Bayesian tree and the Bayesian posterior probability values in tree.

**Additional file 6.** The *SEP* genes in Bayesian tree and the Bayesian posterior probability values in tree.

**Additional file 7.** The *AGL6/AGL13* genes in Bayesian tree and the Bayesian posterior probability values in tree.

## Abbreviations

AG: *AGAMOUS*
*AGL6*: *AGAMOUS*-*LIKE 6*
*AP*: *APETALA*
BEAST: Bayesian evolutionary analysis by sampling trees
BLAST: Basic Local Alignment Search Tool
C-domain: C-terminal domain
*DEF*: *DEFICIENS*
*GLO*: *GLBOSA*
I-domain: intervening domain
K-domain: keratin-like domain
M-domain: MADS-domain
*MCM1*: *minichromosome maintenance*-*1*
MRCA: most recent common ancestor
MUSCLE: MUltiple Sequence Comparison by Log-Expectation
MYA: million years ago
NCBI: National Center for Biotechnology Information database
*Nenu*: *Nelumbo*
non-syn: non-synonymous
PANTHER: Protein ANalysis THrough Evolutionary Relationships
PGDD: Plant Genome Duplication Database
*PI*: *PISTILLATA*
*SEP*: *SEPALLATA*
*SHP*: *SHATTERPROOF*
SMART: Simple Modular Architecture Research Tool
*SRF*: *serum response factor*
*STK*: *SEEDSTICK*
syn: synonymous
TFs: transcription factors
*TM6*: tomato MADS-box gene 6
