# Supplementary material for: Origination and selection of ABCDE and AGL6 subfamily MADS-box genes in gymnosperms and angiosperms
Source: Biol Res. 2019 Apr 24;52:25. doi: 10.1186/s40659-019-0233-8 (PMC6480507; doi:10.1186/s40659-019-0233-8)
Supplement: Supplementary file 1 — Additional file 1. Sequence numbers of 27 plant species. [file 40659_2019_233_MOESM1_ESM.docx]

**Table S1. Sequence numbers of 27 plant species**

| **No** | **Species** | ***AP1*** | ***AP3/PI*** | ***AG/SHP/STK*** | ***SEP*** | ***AGL6/AGL13*** |
| --- | --- | --- | --- | --- | --- | --- |
| 1 | *Gnetum gnemon* | － | *GGM2* | *GGM3* | － | *GGM9*  *GGM11* |
| 2 | *Picea abies* | － | － | *PaMADS1* | － | *PaMADS8*  *PaMADS10*(*) |
| 3 | *Podocarpus macrophyllus* | － | － | － | － | *PmaMADS13* |
| 4 | *Wollemia nobilis* | － | － | － | － | － |
| 5 | *Sciadopitys verticillata* |  | － | － | － | － |
| 6 | *Taxus baccata* | － | － | *TbAG* | － | *TbAGL6* |
| 7 | *Cryptomeria japonica* | － | － | － | － | *CjMADS8*(*)  *CjMADS14* |
| 8 | *Ginkgo biloba* | － | *GbMADS4*  *GbMADS9* | *GBM5*  *GbMADS2*(*) | － | *GbMADS1*  *GbMADS8* |
| 9 | *Amborella trichopoda* | *ERN17823* | *ERM96348*  *ERN20181*  *ERN01839*  *AAR06649*  *AAR06677*  *AAR06678*  *BAD42443*  *BAD42444* | *ERN14157*  *AAY25577* | *ERN09875*  *ERN17839* | *ERM96536* |
| 10 | *Musa accuminata* | *MaMADS7* | *MaMADS6p*  *MaMADS14*  *MaMADS71*  *MaMADS88* | *MaMADS5P*  *MaMADS12*  *MaMADS57* | *MaMADS1p*  *MaMADS2p* | *MaMADS3p*  *MaMADS33*  *MaMADS55*  *MaMADS65*  *MaMADS70* |
| *11* | *Oryza sativa* | *OsMADS14*  *OsMADS15*  *OsMADS18*  *OsMADS20* | *OsMADS2*  *OsMADS4*  *OsMADS16* | *OsMADS3*  *OsMADS13*  *OsMADS21*  *OsMADS58* | *OsMADS1*  *OsMADS5*  *OsMADS7*  *OsMADS8*  *OsMADS34* | *OsMADS17*  *OsMADS6* |
| *12* | *Zea mays* | *ZmMADS8*  *ZmMADS15*  *ZmMADS16*(*) | *ZmMADS20*  *ZmMADS60* | *ZmMADS22*  *ZmMADS23*  *ZmMADS42* | *ZmMADS4p*  *ZmMADS6*(*)  *ZmMADS7*(*) | *ZmMADS33* |
|  |  |  |  |  |  |  |
|  |  | *ZmMADS3P* |  | *ZmMADS36* | *ZmMADS10* |  |
|  |  | *ZmMADS25*(*)  *ZmMADS34*  *ZmMADS50* |  | *ZMM1* | *ZmMADS14*  *ZmMADS 35*  *ZMM3*  *ZMM7*  *ZMM8* |  |
| *13* | *Phalaenopsis aphrodite* | *PATC145405*  *PATC154931* | *PATC138350*  *PATC133864*  *PATC152852*  *PATC154853*  *PATC240636* | *PATC052371*  *PATC138585*  *PATC155109* | *PATC138540*  *PATC141808*  *PATC152066* | *PATC154379*  *PATC138772* |
| *14* | *Solanum lycopersicum* | N | *TM6* | N | N | N |
| *15* | *Solanum tuberosum* | *StMADS7*  *StMADS12*  *StMADS20*  *StMADS55*  *StMADS85*  *StMADS98*  *StMADS121*  *StMADS164*  *StMADS176*  *StMADS181* | *StMADS54*  *StMADS61*  *StMADS86*  *StMADS112*  *StMADS145*  *StMADS153*  *StMADS253* | *StMADS34*  *StMADS71*  *StMADS84*  *StMADS111*  *StMADS114*  *StMADS130*  *StMADS146*  *StMADS155*  *StMADS178* | *StMADS14*  *StMADS137*  *StMADS172*  *StMADS188* | *StMADS165* |
| *16* | *Vitis vinifera* | *VvAP1*  *VvFUL L.*  *VvFUL* | *VvAP3_1*  *VvAP3_2*  *VvPI* | *VvAG1*  *VvAG2*  *VvAG3* | *VvSEP1*  *VvSEP3*  *VvSEP4* | *VvAGL6*  *VvMADS17*(*) |
| *17* | *Citrus sinensis* | *CsiMADS84*  *CsiMADS86* | *CsiMADS33*  *CsiMADS34*  *CsiMADS35*  *CsiMADS41*  *CsiMADS42*  *CsiMADS43*  *CsiMADS46* | *CsiMADS3*  *CsiMADS82* | *CsiMADS1*  *CsiMADS4* | *CsiMADS2*  *CsiMADS37* |
|  |  |  |  |  |  |  |
| *18* | *Carica papaya* | *CpMADS11*  *CpMADS12*  *CpMADS20* | *CpMADS22*  *CpMADS23*  *CpMADS24* | *CpMADS1*  *CpMADS9* | *CpMADS2*  *CpMADS3*  *CpMADS4* | *CpMADS5* |
|  |  |  |  |  | *CpMADS6* |  |
| *19* | *Arabidopsis thaliana* | *AT1G69120* | *AT3G54340*  *AT5G20240* | *AT2G42830*  *AT3G58780*  *AT4G09960*  *AT4G18960* | *AT1G24260*  *AT2G03710*  *AT3G02310*  *AT5G15800* | *AT2G45650*  *AT3G61120* |
| *20* | *Malus domestica* | *MdMADS2p*  *MdMADS5p*  *MdMADS24*  *MdMADS138* | *MdMADS13P*  *MdMADS31*  *MdMADS64*  *MdMADS65*  *MdMADS99*  *MdMADS105*  *MdMADS121*  *MdMADS124*  *MdMADS127*  *MdMADS131*  *MdMADS134*  *MdMADS139*  *MdMADS151* | *MdMADS14p*  *MdMADS21*  *MdMADS30*  *MdMADS59*  *MdMADS126*  *MdMADS170*  *MdMADS172* | *MdMADS8p*  *MdMADS74*  *MdMADS89*  *MdMADS109*  *MdMADS122*  *MdMADS177* | *MdMADS46* |
| *21* | *Cucumis sativus* | *CsMADS07*  *CsMADS08*  *CsMADS09* | *CsMADS21*  *CsMADS23* | *CsMADS24*(*)  *CsMADS44*(*)  *CsMADS45*(*) | *CsMADS01*  *CsMADS02*  *CsMADS03*  *CsMADS04* | *CsMADS05*  *CsMADS06* |
| *22* | *Glycine max* | *Gm MADS24*  *Gm MADS25*  *Gm MADS26*  *Gm MADS27*  *Gm MADS28*  *Gm MADS29*  *Gm MADS30*  *Gm MADS31* | *GmMADS4*  *GmMADS5*  *GmMADS6*  *GmMADS7*  *GmMADS8*  *GmMADS9*  *GmMADS10*  *GmMADS110* | *GmMADS1*  *GmMADS2*  *GmMADS3*  *GmMADS38*  *GmMADS39*  *GmMADS74* | *GmMADS14*  *GmMADS16*  *GmMADS17*  *GmMADS18*  *GmMADS19*  *GmMADS33*  *GmMADS35*  *GmMADS37* | *GmMADS21*  *GmMADS22*  *GmMADS23*  *GmMADS34*  *GmMADS36*  *GmMADS91*(*)  *GmMADS165*(*) |
|  |  | *Gm MADS32*  *Gm MADS69* | *GmMADS121*  *GmMADS133*  *GmMADS147*  *GmMADS175* |  | *GmMADS136*  *GmMADS163* |  |
| *23* | *Nelumbo nucifera* | *AGY54940*  *ADD25192*  *ABG49520* | *ADD25193*  *ADD25194*  *ADD25195*  *ABE11602* | *ADD25188* | *ADD25190*  *ADD25191* | *ADD25189* |
| *24* | *Populus trichocarpa* | *PtMADS16*  *PtMADS33*  *PtMADS39*  *PtMADS50* | *PtMADS10*  *PtMADS11*  *PtMADS22*  *PtMADS25*(*)  *PtMADS30*  *PtMADS38*  *PtMADS45* | *PtMADS18*  *PtMADS34*(*)  *PtMADS43*  *PtMADS51* | *PtMADS6*  *PtMADS13*  *PtMADS17*  *PtMADS31* | *PtMADS37*(*)  *PtMADS40*  *PtMADS46*(*) |
| *25* | *Linum usitatissimum* | *LuMADS17*  *LuMADS24*  *LuMADS31*  *LuMADS60*  *LuMADS75*  *LuMADS78*  *LuMADS92*  *LuMADS107* | *Lu MADS34*  *LuMADS67*  *LuMADS69*  *LuMADS74*  *LuMADS94*  *LuMADS117*  *LuMADS120* | *LuMADS19*  *LuMADS101*  *LuMADS110*  *LuMADS119*  *LuMADS121* | *LuMADS7*  *LuMADS9*  *LuMADS23*  *LuMADS42*  *LuMADS50*  *LuMADS77*  *LuMADS82*  *LuMADS86*  *LuMADS91*  *LuMADS106*  *LuMADS112* | *LuMADS55*  *LuMADS108* |
| *26* | *Ricinus communis* | *RcMADS14*  *RcMADS15*  *RcMADS17* | *RcMADS30*  *RcMADS33*  *RcMADS34* | *RcMADS1*  *RcMADS2*  *RcMADS3* | *RcMADS4*  *Rc MADS5*  *Rc MADS6* | *RcMADS12*  *RcMADS13* |
| *27* | *Manihot esculenta* | *MeMADS21*  *MeMADS22*  *MeMADS23*  *MeMADS26*  *MeMADS28* | *MeMADS45*  *MeMADS46*  *MeMADS47*  *MeMADS48*  *MeMADS52* | *MeMADS3*  *MeMADS4*  *MeMADS5*  *MeMADS12* | *MeMADS1*  *MeMADS7*  *MeMADS8*  *MeMADS9*  *MeMADS14* | *MeMADS15* |
|  | total | 74 | 101 | 75 | 83 | 48 |

Asterisks(*) show newly discovered the sequences in this study. The “N” means uncollected related sequence and the dash mark(-) means unrelated sequence in this study.
