# Supplementary material for: Origination and selection of ABCDE and AGL6 subfamily MADS-box genes in gymnosperms and angiosperms
Source: Biol Res. 2019 Apr 24;52:25. doi: 10.1186/s40659-019-0233-8 (PMC6480507; doi:10.1186/s40659-019-0233-8)
Supplement: Supplementary file 3 — Additional file 3. The AP1 genes in Bayesian tree and the Bayesian posterior probability values in tree. [file 40659_2019_233_MOESM3_ESM.docx]

**
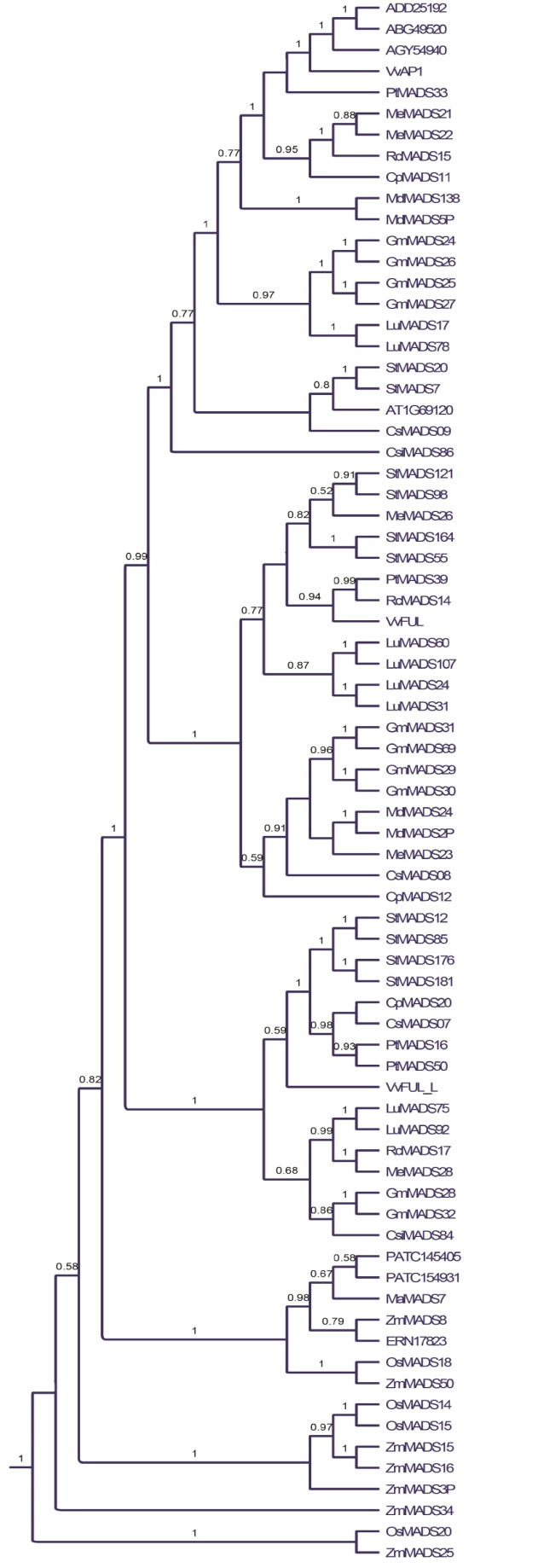
**

**Additional file 3:** The *AP1* genes in Bayesian tree and the Bayesian posterior probability values in tree.
