# Supplementary material for: Origination and selection of ABCDE and AGL6 subfamily MADS-box genes in gymnosperms and angiosperms
Source: Biol Res. 2019 Apr 24;52:25. doi: 10.1186/s40659-019-0233-8 (PMC6480507; doi:10.1186/s40659-019-0233-8)
Supplement: Supplementary file 5 — Additional file 5. The AG/SHP/STK genes in Bayesian tree and the Bayesian posterior probability values in tree. [file 40659_2019_233_MOESM5_ESM.docx]

**
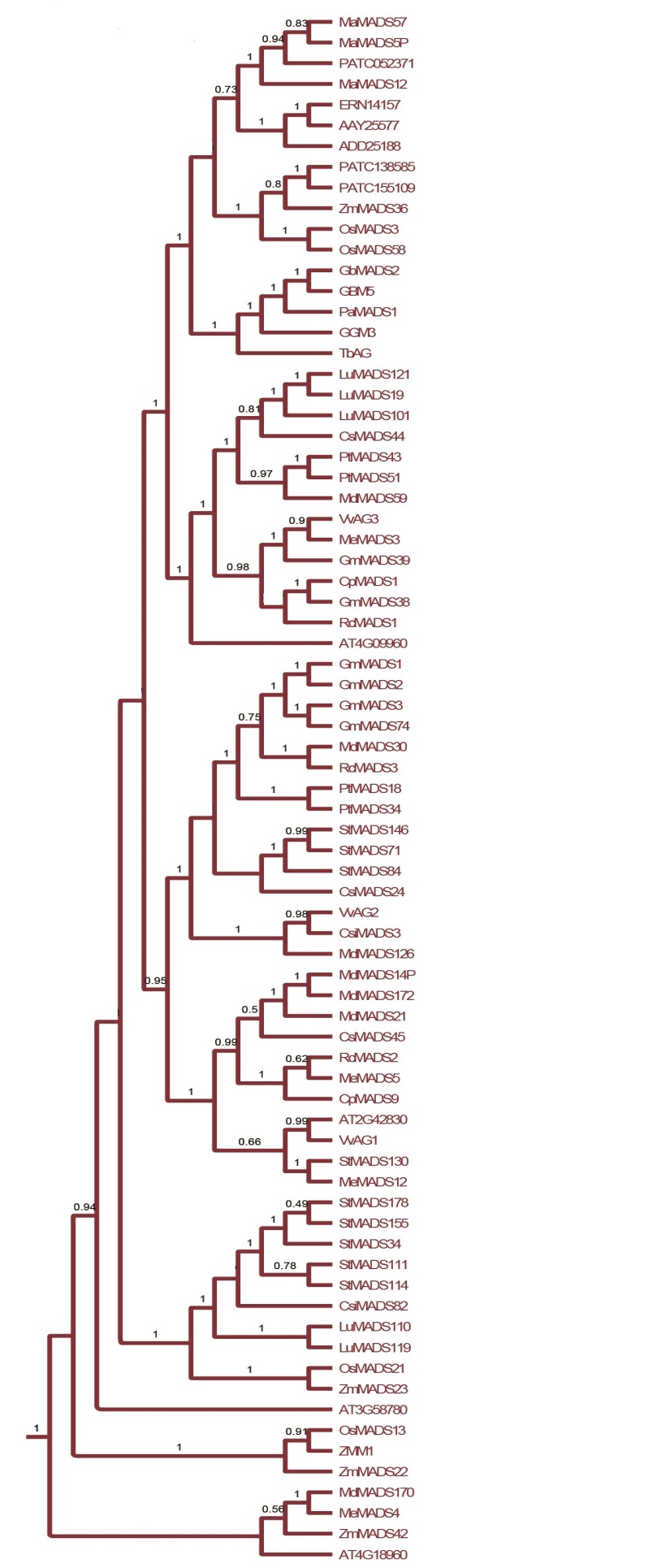
**

**Additional file 5:** The *AG/SHP/STK* genes in Bayesian tree and the Bayesian posterior probability values in tree.
